# Development of RNA Aptamers That Inhibit the RNA-Dependent RNA Polymerase Activity of SARS-CoV-2 Strains In Vitro

**DOI:** 10.3390/ijms262211177

**Published:** 2025-11-19

**Authors:** Chaewon Song, Seong-Wook Lee

**Affiliations:** 1 Department of Bioconvergence Engineering, Research Institute of Advanced Omics, Dankook University, Yongin 16890, Republic of Korea; chaewon37@dankook.ac.kr; 2 R&D Center, Rznomics Inc., Seongnam 13486, Republic of Korea

**Keywords:** SARS-CoV-2, NSP12, RNA aptamer, RNA-dependent RNA polymerase

## Abstract

The continuous emergence of SARS-CoV-2 variants with enhanced transmissibility and immune escape capability underscores the urgent need for mutation-independent anti-viral strategies. SARS-CoV-2 non-structural protein 12 (NSP12), which encodes the RNA-dependent RNA polymerase (RdRp), is an essential component of the viral replication complex and represents a highly conserved target for therapeutic intervention. In this study, we developed RNA aptamers, composed of 2′-hydroxyl nucleotides or 2′-fluoro pyrimidines, targeting NSP12 using the SELEX (Systematic Evolution of Ligands by EXponential enrichment) approach. SELEX was performed with purified NSP12 protein derived from the Omicron variant, leading to the identification of aptamer candidates with high binding ability. RNA–protein pull-down assays confirmed binding between representative aptamers and NSP12 with high affinity. Competition assays supported binding specificity between aptamers and NSP12. Of note, functional evaluation using a primer extension assay revealed that the aptamers effectively inhibited NSP12 RdRp activity in vitro. Furthermore, the aptamers consistently bound to and inhibited NSP12 variants from wild-type, Alpha, Delta, and Omicron strains. These results suggest that the selected RNA aptamers are potential as broad-spectrum inhibitors targeting a conserved region of NSP12 and may serve as a promising platform for the development of anti-viral agents against current and emerging SARS-CoV-2 variants, as well as other RNA viruses.

## 1. Introduction

Coronavirus disease 2019 (COVID-19) caused a global pandemic, and its causative agent has been identified as severe acute respiratory syndrome coronavirus 2 (SARS-CoV-2) [1]. Although the global pandemic has subsided, the ongoing potential for viral mutations and localized resurgences underscores the necessity for long-term strategies to mitigate the emergence of novel viral pathogens [2]. SARS-CoV-2 is a positive-sense single-stranded RNA virus belonging to the family Coronaviridae, subfamily Orthocoronavirinae, genus Betacoronavirus, and subgenus Sarbecovirus [3]. The viral genome is approximately 30 kb in length and comprises 14 open reading frames (ORFs) [4]. Approximately two-thirds of the genome encodes 16 non-structural proteins (NSP1–NSP16), while the remaining portion encodes 9 accessory proteins and 4 structural proteins [5].

The replication of SARS-CoV-2 begins with the translation of ORF1a and ORF1b following virus entry into the host cell. These genes are translated into a large polyprotein, which is subsequently cleaved by viral proteases to yield the individual non-structural proteins (NSPs) [5]. Among these, NSP12 functions as an RNA-dependent RNA polymerase (RdRp) and serves a central role in the replication and transcription of the viral genome [6]. NSP12 consists of 932 amino acids and comprises three domains: an N-terminal nidovirus RdRp-associated nucleotidyltransferase (NiRAN) domain, an interface domain, and a C-terminal RdRp domain [5]. The interface domain serves as a structural bridge between NiRAN and RdRp domains, and the RdRp domain contains a catalytic center with an SDD motif (residues 759–761). Notably, the RdRp of SARS-CoV-2 also possesses a conserved GDD motif (residues 823–825), which is commonly found in other viral RdRps, such as hepatitis C virus (HCV) NS5b and poliovirus 3D pol, and is essential for enzymatic catalysis [7].

Since the emergence of the original wild-type SARS-CoV-2, multiple variants have successively arisen, including Alpha, Beta, Gamma, and Delta, followed by the global dominance of Omicron variants belonging to the BA lineage. Among the Omicron subvariants, JN.1 was designated as a variant of interest (VOI) by the World Health Organization (WHO) in late 2023. More recently, additional sublineages such as KP.3, XEC, LP.8.1, NB.1.8.1, and XFG have continued to appear [8,9,10]. Through these mutations, SARS-CoV-2 can acquire genetic changes that enhance infectivity, immune evasion, and viral load, potentially compromising the effectiveness of existing treatments and vaccines [11,12,13]. The NSP12 protein, a core enzyme responsible for viral replication, harbors the P323L mutation, which has been consistently observed since the emergence of the Alpha variant. Additionally, the G671S mutation has been identified in variants such as Delta, BA.2, and XBB. In the case of JN.1, only the G671S mutation has been reported, whereas Omicron BA.2 possesses both P323L and G671S mutations [14]. These mutations have been reported to increase viral replication and transmissibility [15], underscoring the need to develop novel antiviral agents that target observed molecular regions capable of maintaining efficacy across diverse variants.

RNA aptamers are short single-stranded nucleic acids capable of forming stable secondary structures and exhibiting high binding affinity and specificity toward their target molecules [16,17]. Compared to antibodies, RNA aptamers present several advantages as therapeutic agents, including lower production costs, ease of chemical synthesis, and minimal immunogenicity [18]. Of note, high-affinity aptamers can be selected through the SELEX (Systematic Evolution of Ligands by Exponential Enrichment) process, which involves iterative rounds of selection performed entirely in vitro [19,20,21].

Most studies on aptamers targeting SARS-CoV-2 have focused on DNA aptamers, whereas investigation into RNA aptamers remain relatively limited. Reported RNA aptamers have largely targeted structural proteins, such as spike or nucleocapsid proteins. For instance, RNA aptamers have been developed to bind the spike protein and inhibit viral entry [22], or to recognize the nucleocapsid protein for diagnostic purposes [23]. Moreover, RNA aptamers have been utilized in rapid detection platforms employing fluorescent signal amplification [24], as well as in studies examining binding to multiple spike protein variants [25]. Recently, RNA aptamers capable of binding the RdRp of SARS-CoV-2 have been reported, demonstrating inhibition of polymerase activity in vitro [26]. However, these aptamers were derived from unmodified RNA sequences and were evaluated only against the wild-type (WT) RdRp, without assessing their binding properties to variant forms.

In this study, we aimed to identify RNA aptamers, composed of 2′-hydroxyl nucleotides or 2′-fluoro pyrimidines, that target NSP12, a key replication enzyme of SARS-CoV-2, and to evaluate their binding characteristics and polymerase inhibitory activities. SELEX was performed using purified NSP12 protein derived from the Omicron variant, and high-specificity aptamer candidates were obtained through iterative selection rounds. Binding interactions between the selected aptamers and NSP12 were confirmed by RNA–protein pull-down assays, and binding affinity was quantitatively assessed through the determination of dissociation constants (Kd values). In addition, primer extension assays demonstrated the ability of the aptamers to inhibit polymerase activity, and competition assays further verified their binding specificity. Of note, comparative analysis across NSP12 variants from multiple SARS-CoV-2 variants, including wild-type, Alpha, Delta, and Omicron, revealed that the selected aptamers exhibited consistent binding and inhibitory activity across all tested variants.

Both classes of RNA aptamers, composed of 2′-hydroxyl nucleotides or 2′-fluoro pyrimidines, specifically bound to SARS-CoV-2 NSP12. These aptamers demonstrated high-affinity binding and effectively inhibited NSP12 enzymatic activity irrespective of viral variant, suggesting their potential as foundational candidates for the development of broad-spectrum anti-viral agents.

## 2. Results

### 2.1. Expression and Purification of NSP12(P323L/G671S) and RNA Aptamer Selection by SELEX

**Figure 1:**
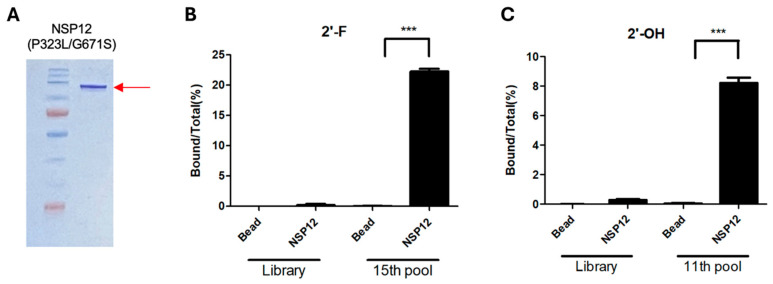
SDS-PAGE Analysis of NSP12(P323L/G671S) and binding evaluation of the selected RNA aptamer pools. (**A**) NSP12(P323L/G671S) protein containing a SUMO tag was expressed in *E. coli* BL21 (DE3) and purified by affinity chromatography. SDS-PAGE analysis showed a prominent band at approximately 125 kDa (red arrow), corresponding to the expected molecular weight of SUMO-tagged NSP12. (**B**) Binding of the 15th-round 2′-fluoro-modified (2′-F) RNA aptamer pools to NSP12(P323L/G671S). The initial library and bead-only groups served as negative controls. (**C**) Binding of the 11th-round 2′-hydroxyl (2′-OH) RNA aptamer pools to NSP12(P323L/G671S). Results are means ± SDs. *** *p* < 0.001 (by a two-tailed Student’s *t*-test).

SARS-CoV-2 NSP12 protein is typically expressed in an insoluble form in *E. coli*. To improve its solubility, an N-terminal His–SUMO-tagged expression vector (Addgene plasmid #169188) was utilized, as this system has been reported to enhance protein folding and solubility [27]. An induction test was performed to optimize expression and the highest yield was obtained with 0.4 mM IPTG at 16 °C for 16 h. Large-scale expression was conducted using these conditions, SDS-PAGE analysis of the purified protein showed a distinct band of ~125 kDa (Figure 1A), corresponding to the expected molecular weight of NSP12 including the His–SUMO tag. The protein used in this study was derived from the Omicron variant, and thus, harbored the P323L and G671S mutations. Accordingly, it was designated NSP12(P323L/G671S). The purified protein was quantified using the Bradford assay and subsequently utilized in SELEX and binding assays.

The purified NSP12(P323L/G671S) protein was used to select RNA aptamers by SELEX. The selection was performed using an RNA library containing a 40-nucleotide randomized region. Nonspecific binders were eliminated by pre-clearing with Ni-NTA magnetic beads prior to incubation with the target protein. RNA molecules specifically bound to NSP12(P323L/G671S) were recovered, reverse-transcribed, PCR-amplified, and transcribed in vitro to generate RNA pools for subsequent rounds. To enhance aptamer diversity and assess potential for downstream applications, both unmodified 2′-hydroxyl (2′-OH) RNAs or chemically stabilized 2′-fluoro pyrimidine (2′-F) RNAs were independently subjected to SELEX. Selection stringency was progressively increased by adding competitor yeast tRNA and reducing the target protein concentration in later rounds. This strategy enabled the enrichment of high-affinity aptamers with improved specificity and stability toward NSP12(P323L/G671S).

Binding enrichment of the selected RNA pools was evaluated through RT-PCR–based confirmation assays. For the 2′-F RNA pools, binding activity was compared between the initial RNA library and the pool RNAs obtained after 15 rounds of SELEX. The 15th-round pool exhibited ~22% binding to NSP12(P323L/G671S), significantly higher than the initial library and the bead-only control (Figure 1B), indicating successful enrichment of high-affinity binders. Similar analysis showed that the 2′-OH RNA pools obtained after 11 SELEX rounds displayed ~8% binding to NSP12 (Figure 1C), confirming the enrichment of target-specific aptamers within this pool.

### 2.2. Grouping and Comparative Binding Evaluation of SELEX-Enriched Aptamers

To identify individual aptamer sequences, RT-PCR was performed using the 15th-round 2′-F RNA pools or the 11th-round 2′-OH RNA pools as templates, followed by T-blunt cloning and sequencing. Sequences derived from the 2′-F and 2′-OH pools are designated the “CV-F” and “CV-OH” groups, respectively, and serial identifiers (e.g., CV-F1, CV-OH1) are assigned for clarity.

Sequence analysis classified the 2′-F pools into five sequence groups and the 2′-OH pools into four groups based on sequence similarity and predicted secondary structure (Table 1). Representative aptamers from each group-CV-F1 to CV-F5 and CV-OH1 to CV-OH4-were selected for subsequent binding analysis.

**Figure 2:**
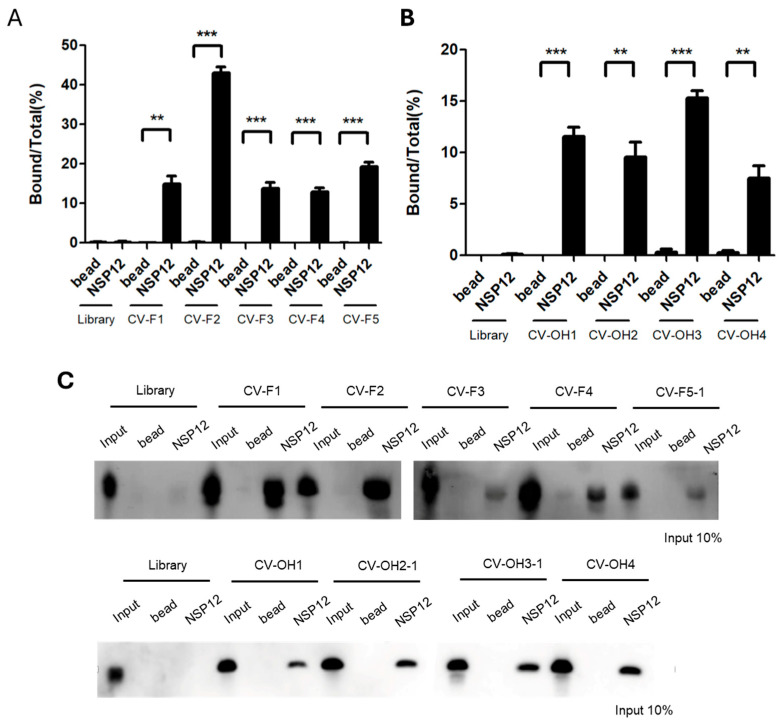
Binding abilities of SELEX-enriched RNA aptamers to SARS-CoV-2 NSP12(P323L/G671S). (**A**) Quantification of bound 2′-F RNA aptamers by qRT-PCR. Among the tested candidates, CV-F2 showed the highest binding to NSP12(P323L/G671S) (~40%). (**B**) Quantification of bound 2′-OH RNA aptamers by qRT-PCR. CV-OH3 showed the highest binding (~17%). (**C**) RNA–protein pull-down assay. Three lanes were applied: The input lanes represent 10% of the total RNA used in each reaction, which was directly loaded onto gel without any binding or washing steps. This lane served as an internal control for RNA detection and quantitative comparison. The bead lanes included magnetic beads without NSP12 protein and served as negative controls to assess nonspecific binding. The NSP12 lanes included RNA aptamers incubated with SARS-CoV-2 NSP12(P323L/G671S) protein to evaluate specific binding. (**Upper panel**) Results of 2′-F aptamers. Strong binding signals were observed for CV-F2 and CV-F1, whereas weaker signals were observed for CV-F3 to CV-F5-1. (**Lower panel**) Results of 2′-OH aptamers. Moderate binding signals were observed for all aptamer clones. Results represent means ± SDs (n = 3). Statistical significance was assessed using a two-tailed Student’s *t*-test: ** *p* < 0.01, *** *p* < 0.001.

Binding affinities to NSP12(P323L/G671S) were assessed by qRT-PCR quantification and RNA–protein pull-down assays. Among the 2′-F aptamers, CV-F2 had the highest binding activity with a bound-to-total RNA ratio of ~40% (Figure 2A), whereas CV-F1, CV-F3, CV-F4, and CV-F5-1 showed relatively weaker binding. For the 2′-OH aptamers, qRT-PCR analysis revealed moderate binding across all candidates, with CV-OH3-1 exhibiting the highest affinity (~17%) (Figure 2B). These results were corroborated by pull-down assays, which showed CV-F2 and CV-F1 had strong binding ability, while the other 2′-F aptamer clones and all 2′-OH RNA aptamer clones had moderate binding ability (Figure 2C).

Collectively, these results indicate that CV-F2 and CV-OH3-1 possess the strongest binding affinities within their respective groups, and thus, these two aptamers were selected for further functional characterization.

### 2.3. Affinity and Specific Binding of Selected RNA Aptamers

**Figure 3:**
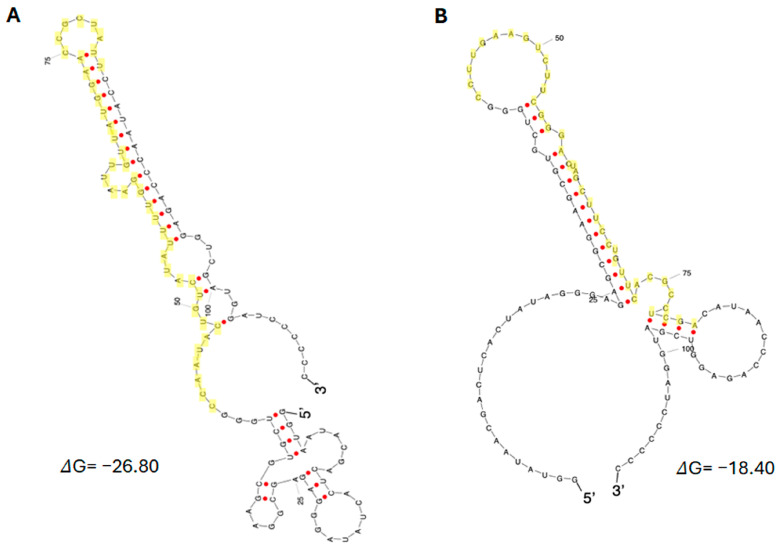
Predicted secondary structures of the representative aptamers. (**A**) CV-F2 (2′-F RNA aptamer). (**B**) CV-OH3-1 (2′-OH RNA aptamer). Structures were predicted using sfold. Yellow regions indicate selection-enriched sequences identified by SELEX. Base pairs are indicated as red dots.

The predicted secondary structures of the representative aptamers CV-F2 and CV-OH3-1 are shown in Figure 3. Minimum free energy predictions using the sfold web server (https://sfold.wadsworth.org/cgi-bin/srna.pl (accessed on 14 November 2025)) indicated each aptamer forms a distinct stem–loop conformation. The regions highlighted in yellow correspond to sequences enriched during SELEX, which are presumed to be critical for specific interactions with NSP12.

**Figure 4:**
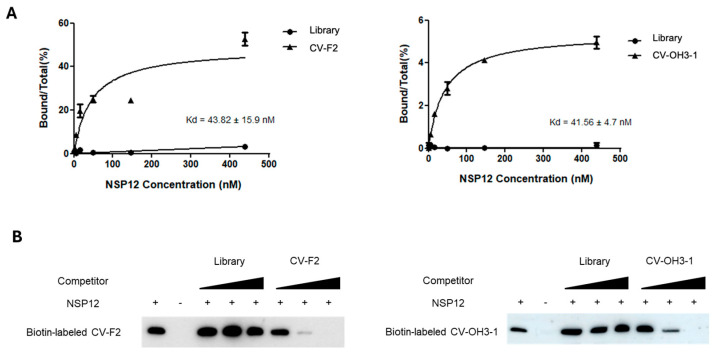
Biding affinity and specificity of the representative aptamers. (**A**) Binding curves of CV-F2 and CV-OH3-1 to NSP12(P323L/G671S) as determined using a qRT-PCR–based assay. Aptamers were incubated with increasing concentrations of NSP12 protein, and the bound fractions were quantified. Both CV-F2 and CV-OH3-1 exhibited high binding affinity with dissociation constants (Kd) of 43.82 ± 15.9 nM and 41.56 ± 4.7 nM, respectively. In contrast, the RNA library control showed negligible binding. (**B**) RNA–protein pull-down competition assay using biotin-labeled aptamers. Increasing concentrations of unlabeled competitor RNAs (either the same aptamer or non-specific library RNA) were added to assess binding specificity. The bindings of biotin-labeled CV-F2 and CV-OH3-1 were effectively inhibited by their unlabeled counterparts but remained unaffected by library RNA, indicating specific interactions with NSP12.

To observe biochemical binding strength of the aptamers, quantitative evaluation of the binding affinity of the selected aptamers toward SARS-CoV-2 NSP12(P323L/G671S) was performed using qPCR-based Kd analysis (Figure 4A). Experiments were conducted at a fixed aptamer concentration and gradually increasing concentrations of NSP12(P323L/G671S) protein. After each binding reaction, the amount of RNA bound to the beads was quantified by qPCR. The relative binding ratios were calculated to generate binding curves.

The 2′-F aptamer CV-F2 exhibited a binding affinity with a dissociation constant (Kd) of 43.82 ± 15.9 nM for NSP12(P323L/G671S), whereas the 2′-OH aptamer CV-OH3-1 had a Kd of 41.56 ± 4.7 nM (Figure 4A), indicating both aptamers possess high binding affinity in the nanomolar range toward the target protein. In contrast, the RNA library used as a negative control did not generate a specific binding curve despite increasing protein concentrations, and thus, no Kd could be determined. This suggests that the library RNA exhibits nonspecific or weak binding to NSP12.

Binding specificity was further evaluated using an RNA–protein pull-down competition assay (Figure 4B). Biotin-labeled aptamers were incubated with NSP12(P323L/G671S) in the presence of either unlabeled aptamers of the same sequence or nonspecific library RNA. The amount of pulled-down biotin-labeled aptamer were then quantified (Figure 4B). The addition of unlabeled library RNA did not affect the band intensity, whereas competition with unlabeled aptamers of identical sequence caused a dose-dependent decrease in band intensity.

These results indicate that CV-F2 and CV-OH3-1 have high affinity and specificity toward SARS-CoV-2 NSP12(P323L/G671S) and are potential as promising candidates for the development of anti-viral therapeutics.

### 2.4. Inhibition of NSP12(P323L/G671S) Replication Activity by Selected RNA Aptamers

**Figure 5:**
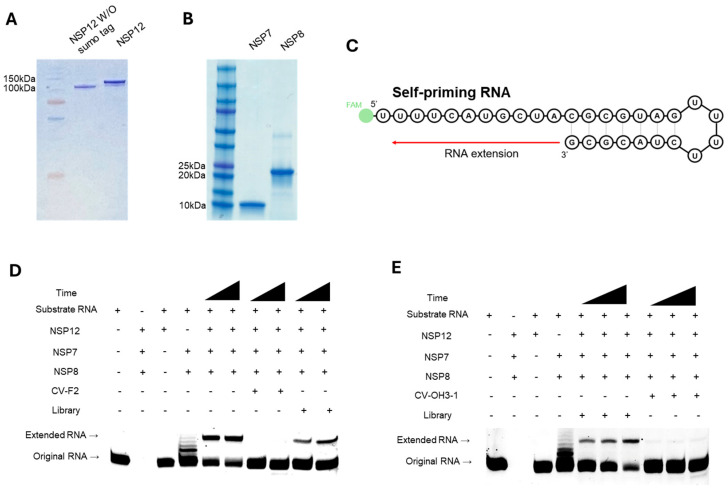
Inhibition of SARS-CoV-2 NSP12(P323L/G671S) replication activity by the selected RNA aptamers. (**A**) SDS-PAGE analysis of purified NSP12(P323L/G671S) protein with or without the SUMO tag. A single band around 100 kDa indicates successful purification. A molecular weight marker was loaded in the leftmost lane. (**B**) SDS-PAGE analysis of NSP7 and NSP8 cofactors, showing distinct bands at ~10 kDa and ~20 kDa, respectively. (**C**) Schematic representation of the self-priming RNA substrate used in the primer extension assay. The RNA substrate included a 5′ FAM label and forms a stem-loop structure that enables self-priming from the 3′ end. Polymerase activity results in RNA extension toward the 5′ end of the substrate, generating a longer RNA product. (**D**,**E**) Primer extension assays were used to evaluate the inhibitory effects of selected RNA aptamers on the polymerase activity of SARS-CoV-2 NSP12(P323L/G671S). Reactions were performed using the self-priming RNA substrate in the presence of NSP12, NSP7, and NSP8 proteins. (**D**) CV-F2 (2′-F RNA aptamer) and (**E**) CV-OH3-1 (2′-OH RNA aptamer) both resulted in a marked reduction in the extended RNA product, indicating effective inhibition of polymerase activity. In contrast, incubation with the initial aptamer libraries did not affect polymerase activity. Components in each gel lane are indicated above each image.

A primer extension assay was used to evaluate the inhibitory effect of the selected aptamer candidates on the polymerase activity of NSP12(P323L/G671S). The purified RNA-dependent RNA polymerase (RdRp) complex of SARS-CoV-2, comprising NSP12, NSP7, and NSP8, was used for this analysis. The SUMO tag-removed NSP12 protein was employed (Figure 5A), and the purified NSP7 and NSP8 proteins were verified by SDS-PAGE (Figure 5B). The polymerization reaction was performed using a self-priming FAM-labeled RNA template (Figure 5C), and extension of the RNA strand was monitored to assess polymerase activity.

The inhibitory capacities of CV-F2 and CV-OH3-1 were then examined. In the presence of either of these aptamers, the signal intensity of the extended RNA band was markedly reduced, indicating that both aptamers inhibited the polymerase activity of NSP12 and interfered with the RNA extension process (Figure 5D,E). In contrast, the RNA library control did not inhibit polymerase activity under the same conditions. These results suggest that CV-F2 and CV-OH3-1 aptamers specifically bind to NSP12 and effectively attenuate the functional activity of the SARS-CoV-2 replication complex, thereby impeding RNA synthesis.

### 2.5. Binding to NSP12 Variants and Inhibition of Their Replication Activity by the Selected RNA Aptamers

**Figure 6:**
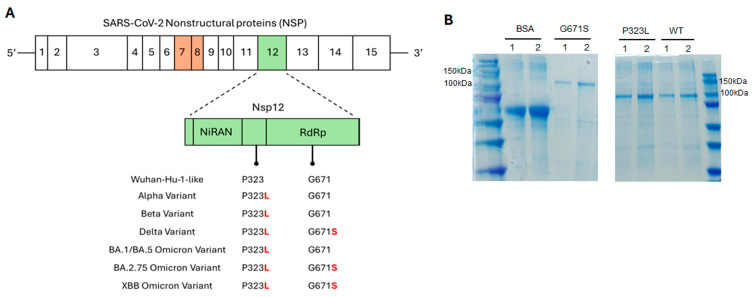
Domain structure of SARS-CoV-2 NSP12 and mutation profiles across variants. (**A**) Schematic representation of domain organization of SARS-CoV-2 non-structural proteins (NSPs), highlighting NSP12, the RNA-dependent RNA polymerase (RdRp). NS12 comprises an N-terminal NiRAN domain, an interface domain, and a C-terminal RdRp domain. Amino acid residues P323 and G671, located within the interface and RdRp domains, respectively, represent frequent mutation hotspots. The mutation profiles of representative SARS-CoV-2 variants are provided below; The P323L is conserved in all major lineages, whereas G671S is found specifically in Delta, BA.2.75, and XBB Omicron variants. (**B**) SDS–PAGE analysis of purified recombinant NSP12 proteins. Wild-type (WT) and mutant NSP12 proteins (P323L and G671S) were expressed and purified from *E. coli.* Lanes 1 and 2 correspond to 5 µL and 10 µL sample loadings, respectively, for each purified NSP12 protein (WT, P323L, and G671S). Bovine serum albumin (BSA), loaded at 1 µg (lane 1) and 2 µg (lane 2), served as a loading control.

To assess the applicability of the selected aptamers across SARS-CoV-2 variants, their binding affinities and inhibitory activities were evauated against NSP12 proteins harboring key mutations. NSP12 functions as a highly conserved viral replicase with NiRAN and RdRp domains, however, amino acid substitutions at positions P323 and G671 have frequently been reported among major SARS-CoV-2 variants (Figure 6A). Specifically, the P323L mutation is characteristic of early variants such as Alpha, Beta, and Delta, whereas the G671S mutation commonly appears in later variants, including Delta, BA.2, and XBB lineages. The Omicron-type NSP12 used in the SELEX process contained both P323L and G671S substitutions, thereby representing a prevalent mutation pattern.

To verify the expression and purity of NSP12 variants used for subsequent binding and inhibition assays, the wild-type (WT) and mutant (P323L and G671S) proteins were expressed and purified, as confirmed by SDS–PAGE (Figure 6B). The G671S substitution alone was included to independently evaluate the aptamer’s effect on the G671S variant of NSP12, although this single G671S has not been observed by itself in any known vial variants.

**Figure 7:**
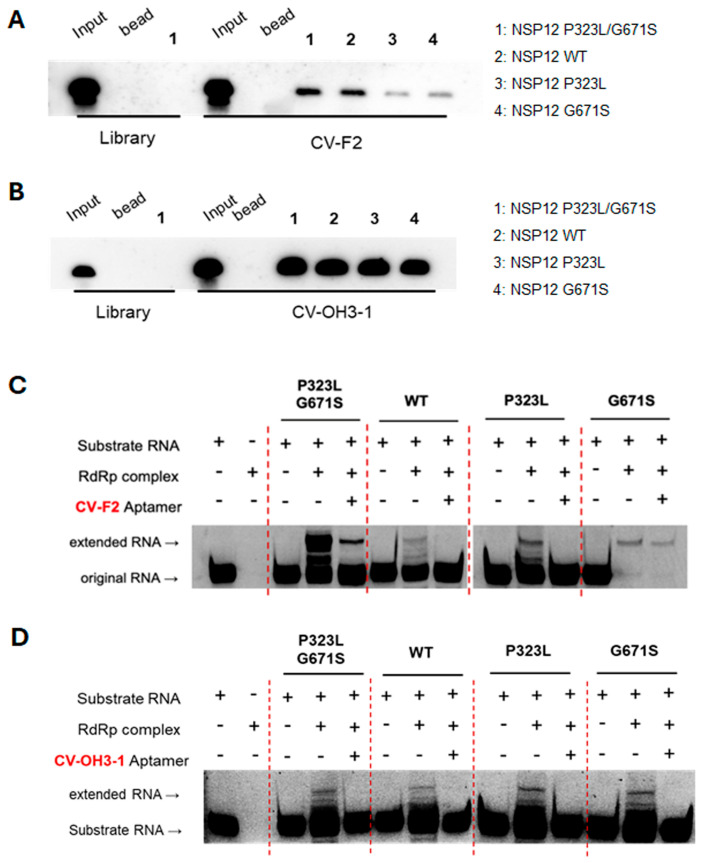
Evaluation of the binding ability and replication inhibition activity of RNA aptamers against various SARS-CoV-2 NSP12 variants. (**A**,**B**) RNA–protein pull-down assays were conducted to examine the binding of biotin-labeled CV-F2 (**A**) and CV-OH3-1 (**B**) aptamers to recombinant NSP12 proteins harboring different mutations (WT, P323L, G671S, or P323L/G671S). RNA–protein complexes were isolated using magnetic beads, and detected by streptavidin-based chemiluminescence. The initial RNA library served as a negative control. (**C**,**D**) Primer extension assays were conducted to assess inhibitory effect of CV-F2 (**C**) and CV-OH3-1 (**D**) aptamers on the polymerase activity of the reconstituted RdRp complex (NSP12 + NSP7 + NSP8). Using a self-priming RNA substrate, each aptamer significantly reduced synthesis of extended RNA product across all forms of NSP12 variants, including the wild-type (WT) and single and double mutants, indicating variant-independent inhibition of polymerase activity.

Given the high mutation rate of RNA viruses and the potential impact of such mutations on aptamer–target interactions, the selected aptamers were evaluated for their ability to function across multiple NSP12 variants. To this end, recombinant NSP12 proteins representing the wild-type, single mutants (P323L or G671S), and a double mutant (P323L/G671S) were generated. RNA–protein pull-down assays revealed that both CV-F2 (a 2′-F aptamer) and CV-OH3-1 (a 2′-OH aptamer) specifically bound all tested NSP12 variants, including the wild-type and the mutant forms (Figure 7A,B).

The inhibitory effects on NSP12 RNA polymerase activity were further evaluated using primer extension assays. Both CV-F2 and CV-OH3-1 effectively suppressed RNA polymerase activity when NSP12 was reconstituted with its cofactors NSP7 and NSP8. This inhibitory effect was consistent across the wild-type enzyme as well as the P323L, G671S, and P323L/G671S mutant enzymes (Figure 7C,D). However, the extent of inhibition of RNA extension activity by each aptamer did not always correlate with its binding efficiency, indicating the need for a more detailed investigation into the molecular mechanism by which aptamer binding affects the enzymatic activity of the target. The initial aptamer libraries as controls did not exhibit inhibitory effect under the same conditions.

Collectively, these findings demonstrate that the selected aptamers retain high binding affinity and robust inhibitory activity, regardless of mutation statue in NSP12. This suggests their potential as broad-spectrum anti-viral agents, with activity against the viral RNA polymerase complex across diverse SARS-CoV-2 lineages.

## 3. Discussion

In this study, high-affinity RNA aptamers that bind to and inhibit the activity of the SARS-CoV-2 RNA-dependent RNA polymerase (RdRp) NSP12 protein were identified and functionally validated. Notably, the SELEX process was performed using a mutant form of NSP12 (P323L/G671S) as the target to develop anti-viral aptamers capable of retaining efficacy against emerging viral variants.

The aptamers CV-F2 (2′-F modified RNA) and CV-OH3-1 (2′-OH RNA), identified by SELEX, exhibited dissociation constants (Kd) in the nanomolar range, indicating strong and specific binding affinity for SARS-CoV-2 NSP12 protein. However, further evaluation in virus-infected cellular models is necessary to determine whether this high binding affinity translates into effective antiviral activity. RNA–protein pull-down and competition assays demonstrated that both aptamers possess significantly higher binding specificity compared to the initial RNA library. Binding was competitively inhibited only by the addition of the same unlabeled aptamer sequence, supporting that the interaction with NSP12 is mediated by selective structural complementarity.

Functional analysis using primer extension assays confirmed that CV-F2 and CV-OH3-1 effectively inhibited NSP12 polymerase activity. Importantly, this inhibitory effect was consistently observed not only with the P323L/G671S double mutant NSP12 used during SELEX, but also with the wild-type NSP12 and each of the single mutants (P323L and G671S). This finding underscores the broad applicability of these aptamers, suggesting their efficacy across various NSP12 variants potentially arising from different SARS-CoV-2 strains. As an RNA virus, SARS-CoV-2 exhibits a high mutation rate; the P323L mutation in NSP12 has been consistently maintained across major variants of concern, including the Alpha, Beta, Delta, and Omicron [28,29], while the G671S mutation is highly prevalent, detected in ~97.8% of Delta variants and ~99% of the Omicron XBB subvariant [30]. Comprehensive sequence analyses estimate the frequency of P323L and G671S mutations to be approximately 88.2% and 15.6%, respectively [30]. These mutations have been shown to enhance the stability and enzymatic activity of the RdRp complex, thereby increasing the efficiency of viral replication and transmission [15,31]. This study demonstrates the potential of aptamer-based approach to effectively respond to the mutational diversity of SARS-CoV-2.

Conventional therapeutic strategies against SARS-CoV-2 have primarily relied on antibodies or small molecules, with remdesivir serving as a representative RdRp inhibitor. Nonetheless, emergence of drug-resistant mutations such as E802D in the nsp12 gene of immunocompromised patients during remdesivir treatment has been documented [32]. Moreover, NSP12 mutations pose concerns regarding resistance to RdRp-targeting antivirals including remdesivir and molnupiravir [33,34]. In contrast, aptamers offer several advantages, including structural flexibility, low immunogenicity, ease of synthesis, and chemical stability, making them promising next-generation therapeutic candidates capable of adapting to rapidly evolving viruses [35,36].

Zhang et al. (2025) recently developed UltraSelex, a noniterative aptamer discovery platform that rapidly identifies high-affinity RNA aptamers targeting various proteins, including SARS-CoV-2 RdRp [26]. This method achieves remarkable binding affinities using unmodified RNA sequences within a short time frame, demonstrating its potential as an efficient and time-saving aptamer discovery strategy. In contrast, the present study employed iterative SELEX using both unmodified and 2′-fluoro–modified RNA libraries and comprehensively validated the functional inhibitory effects of selected aptamers on both wild-type and mutant RdRp variants, emphasizing their mutational tolerance and biological relevance. These two approaches can be viewed as complementary rather than competing. Integrating the speed and high-throughput capability of UltraSelex with the functional and variant-specific validation framework demonstrated here could accelerate the development of robust, broad-spectrum antiviral aptamers in the future.

The aptamers identified in this study exhibited high-affinity binding to and effective inhibition of the purified RdRp complex (NSP12/NSP7/NSP8) in vitro, suggesting their potential utility for precise modulation and analysis of viral replication. Furthermore, chemical modifications such as 2′-fluoro are known to enhance in vivo stability, and thereby further increase therapeutic potential of aptamers [22,37,38]. Aptamers could exert antiviral effects as molecular decoys, with intracellular delivery strategies including direct chemical synthesis and delivery of 2′-fluoro RNA aptamers via non-viral vectors, or delivery and expression of 2′-OH RNA aptamers using DNA viral vectors [18].

Nevertheless, this study has several limitations. First, the antiviral activities of the aptamers have not been validated in cellular or animal models, necessitating further studies assess in vivo efficacy and stability. Second, potential off-target binding to other cellular proteins was not directly investigated; however, given that host cells lack RNA-dependent RNA polymerases, off-target effects are expected to be minimal. Nevertheless, cellular toxicity studies are still warranted. Third, structural information elucidating the binding interface between aptamers and the target protein is lacking; future structural biology approaches such as cryo-electron microscopy (cryo-EM) or nuclear magnetic resonance (NMR) should be explored to provide a more precise understanding and means of optimizing the binding mechanism. Such structural insight may also facilitate prediction of mutational escape. Lastly, strategies for enhancing cellular uptake and in vivo delivery of the aptamers, such as conjugation with drug delivery platforms like dendrimers or lipid nanoparticles, should also be considered

In summary, this study successfully identifies highly specific aptamers targeting the SARS-CoV-2 NSP12 (P323L/G671S) protein and demonstrates that they retain strong binding affinity and polymerase inhibitory activity across various NSP12 variants. These findings highlight the potential of aptamers as novel anti-viral platforms against SARS-CoV-2 and its emerging variants. Nevertheless, the direct antiviral efficacy in cellular systems remains to be validated in future investigations using infected cells and pseudovirus models.

## 4. Materials and Methods

### 4.1. NSP12 Protein Purification

The pK27Sumo_His-SUMO-nsp12 (SARS-CoV-2) expression vector (Addgene plasmid #169188; Addgene, Watertown, MA, USA) was transformed into *E. coli* BL21 competent cells. Transformed cells were cultured in 1 L of LB medium supplemented with 10 µg/mL kanamycin and induced with 0.4 mM IPTG at 16 °C for 16 h. After harvesting by centrifugation (4000 rpm, 4 °C), cell pellets were stored at −80 °C overnight. For protein purification, pellets were resuspended in His-SUMO lysis buffer (50 mM Tris-HCl pH 7.5, 500 mM NaCl, 5 mM MgCl_2_, 10% glycerol, 1 mM DTT, 0.1% NP-40, 30 mM imidazole), and treated with lysozyme (100 mg/mL) at 4 °C for 3 h. Cells were then lysed by sonication and centrifuged (13,000 rpm, 4 °C, 1 h). Supernatant was incubated with Ni-NTA agarose beads (Qiagen, Hilden, Germany) for 3 h at 4 °C with rotation and loaded onto a polypropylene column. The column was washed twice with His-SUMO buffer and eluted sequentially with 100 mM, 200 mM, and 400 mM imidazole-containing buffer (50 mM Tris-HCl pH 7.5, 300 mM NaCl, 5 mM MgCl_2_, 10% glycerol, 1 mM DTT, 0.05% NP-40). Eluted fractions were analyzed by SDS-PAGE on a 10% gel. Collected fractions were pooled and dialyzed using a 10,000 MWCO membrane (Cellu-Sep, Dohgil Biotech, Seoul, Republic of Korea) in His-SUMO dialysis buffer (25 mM Tris-HCl pH 7.5, 250 mM NaCl, 5 mM MgCl_2_, 10% glycerol, 0.02% NP-40, 1 mM DTT) at 4 °C overnight, and then dialyzed again for 3 h in fresh buffer. The final purified NSP12 protein was stored at −80 °C.

### 4.2. NSP7 and NSP8 Protein Purification

The pQE-(NSP12)-pcI-ts,ind+-(NSP7-NSP8) vector (Plasmid #160540) was obtained from Addgene and used as a template to individually amplify the NSP7 and NSP8 genes via PCR. The 5′ primer (5′-ATGTCTAAGATGAGTGATGT-3′) and the 3′ primer (5′-TCATTGCAGGGTTGCGC-3′) were used to amplify NSP7, and the 5′ primer (5′-ATGGCTATAGCATCTGAATT-3′) and 3′ primer (5′-TTACTGCAGTTTTACGGCCG-3′) were used to amplify NSP8. Each PCR reaction contained 10 pmol of each primer and 2× CloneAmp HiFi polymerase mix (Takara Bio, Shiga, Japan). The PCR conditions were as follows: 98 °C for 30 s, 58 °C for 15 s, and 72 °C for 10 s, repeated for 35 cycles. The amplified NSP7 and NSP8 fragments were cloned into pET-28a(+) expression vector. Recombinant vectors were transformed into *E. coli* BL21 competent cells. Protein expression was induced with 0.2 mM IPTG at 37 °C for 4 h. After induction, cells were harvested by centrifugation and stored at −80 °C overnight. Cell pellets were lysed by resuspending them in lysis buffer (50 mM Tris-HCl pH 8.0, 500 mM NaCl, 30 mM imidazole, 10% glycerol, 2.5 mM DTT), adding lysozyme (100 mg/mL), and incubating at 4 °C with rotation. Cell disruption was performed by sonication, and the lysate was cleared by centrifugation. The supernatant was incubated with Ni-NTA agarose beads (Qiagen, Hilden, Germany) and loaded onto a polypropylene column. After washing with buffer containing 50 mM imidazole, bound proteins were eluted stepwise using 100, 200, and 400 mM imidazole-containing buffers. Eluted fractions were analyzed by SDS-PAGE (10%) to confirm purity. Proteins were dialyzed overnight at 4 °C using dialysis buffer (50 mM Tris-HCl pH 7.5, 50 mM NaCl, 0.2 mM EDTA, 2.5 mM DTT, 10% glycerol) and then for an additional 3 h in fresh buffer. Final protein samples were stored at −80 °C.

### 4.3. In Vitro Transcription and Purification of 2′-OH and 2-F RNA Libraries

Two types of RNA libraries were prepared for SELEX: a 2′-hydroxyl (2′-OH) RNA library and a 2′-fluoro (2′-F) modified RNA library. Both were transcribed from a randomized N40 DNA library generated by PCR. To synthesize the 2′-OH RNA library, in vitro transcription was performed using 1 µg of DNA template, 10× transcription buffer, 75 mM of each of ATP, UTP, GTP, and CTP, and T7 enzyme mix (MEGAscript™ T7 Transcription Kit, Thermo Fisher Scientific, Waltham, MA, USA). The reaction mixture was incubated at 37 °C for 3 h and then treated with DNase I at 37 °C for 15 min to remove residual DNA. For the 2′-F RNA library, transcription was performed using 1 µg of DNA template, 5 mM of each of 2′-OH ATP and 2′-OH GTP, and 5 mM of each of 2′-F CTP and 2′-F UTP, along with 10× transcription buffer, 10 mM DTT, and 1 µL of T7 RNA polymerase (Durascribe^®^ T7 Transcription Kit, LGC Biosearch Technologies, Novato, CA, USA). The reaction mixture was incubated at 37 °C for 16 h and then subjected to DNase I treatment at 37 °C for 30 min. After transcription, RNA products were purified by phenol–chloroform extraction and ethanol precipitation. The RNA pellet was resuspended in 10 µL of DEPC-treated water, mixed with 2× RNA loading dye, denatured at 95 °C for 5 min, and cooled on ice for 5 min. Samples were resolved by electrophoresis on an 8% denaturing polyacrylamide gel containing 8 M urea at 180 V for 1 h. Target-sized RNA bands were visualized under UV light, excised, and incubated in 500 µL of DEPC-treated water at 37 °C for 3 h to elute the RNA. The eluate was subjected to phenol–chloroform extraction and ethanol precipitation, and the final RNA was resuspended in 20 µL of DEPC-treated water. RNA concentration was measured using a NanoDrop spectrophotometer. The sequence of the RNA library was 5′-GGGAGAGCGGAAGCGUGCUGGGCC-N40-CAUAACCCAGAGGUCGAUGG AUCCCC-3′, where N40 represents equimolar incorporation of each base at each position.

### 4.4. SELEX Procedure

For each selection round, 300 pmol of RNA library was denatured at 95 °C for 5 min and immediately cooled on ice for 5 min. Separately, 20 µL of HisPur Ni-NTA magnetic beads (Thermo Fisher Scientific, Waltham, MA, USA) were washed three times with 400 µL of SELEX binding buffer (30 mM Tris-HCl, pH 7.5, 150 mM NaCl, 1.5 mM MgCl_2_, 2 mM DTT, 1% BSA) supplemented with RNase inhibitor (Labo Pass, Seoul, Republic of Korea). The beads were then resuspended in 200 µL of SELEX buffer and incubated with the denatured RNA at room temperature for 20 min to remove RNA sequences with nonspecific affinity for the beads. After incubation, the RNA–bead mixture was centrifuged and placed on a magnetic rack to collect the supernatant, which contained unbound RNA. This supernatant was incubated with 150 pmol of recombinant NSP12(P323L/G671S) protein at room temperature for 20 min to allow specific RNA–protein complex formation. These complexes were captured by incubation with fresh Ni-NTA beads for 20 min and washed twice with 400 µL of SELEX buffer to remove unbound RNA. Bound RNA was eluted by adding 200 µL of DEPC-treated water, followed by phenol–chloroform extraction and ethanol precipitation, then resuspended in 10 µL of DEPC-treated water. Reverse transcription was performed using 5× reverse transcription buffer, 10 mM dNTPs, 10 pmol of the SEL 3′ primer (5′-GGGGGGATCCATCGACCTCTGGGTTATG-3′), 200 units of reverse transcriptase (OneScript^®^ Plus Reverse Transcriptase, Applied Biological Materials Inc., Richmond, BC, Canada), and RNase inhibitor. The reaction mixture was incubated at 42 °C for 50 min, then at 95 °C for 5 min to deactivate emzymes. The resulting cDNA was amplified by PCR using DNA polymerase (FIREPol^®^ DNA Polymerase, Solis BioDyne, Tartu, Estonia) with SEL 5′ (5′-GGTAATACGACTCACTATAGGGAGAGCGGAAGCGTGCTGGG-3) and SEL 3′ primers. PCR conditions were: 95 °C for 30 s, 58 °C for 30 s, and 72 °C for 30 s, for 20 cycles. Amplified DNA was then transcribed in vitro for the next iterative selection rounds.

### 4.5. qRT-PCR-Based Binding Assay for NSP12(P323L/G671S)

To evaluate the binding activity of enriched RNA pools, 60 fmol of RNA from each of the following pools was used: the 15th-round 2′-F RNA, the 11th-round 2′-OH RNA, and the initial RNA library. Each RNA sample was incubated with 12 pmol of NSP12(P323L/G671S) protein at room temperature. After incubation, 20 µL of Ni-NTA magnetic beads (Thermo Fisher Scientific, Waltham, MA, USA) were added, and the mixture was gently mixed at room temperature for 20 min. The beads were then collected using a magnetic rack and washed twice with 400 µL of SELEX buffer to remove unbound RNAs. Bound RNAs were then eluted with 200 µL of DEPC-treated water, phenol extracted, and precipitated with ethanol. The recovered RNA was resuspended in 10 µL of DEPC-treated water and used for reverse transcription. Reverse transcription was performed using 5× RT buffer, 10 mM dNTPs, 10 pmol of SEL 3′ primer, OneScript Plus reverse transcriptase (Applied Biological Materials Inc., Richmond, BC, Canada), and 0.3 µL of RNase inhibitor. The reaction was incubated at 42 °C for 50 min, followed by enzyme inactivation at 95 °C for 5 min and cooling at 4 °C for 10 min. Real-time PCR was conducted using 25 µL of SensiFAST SYBR Hi-ROX premix (Meridian Bioscience, Cincinnati, OH, USA) and 10 pmol of each SEL 5′ and SEL 3′ primers. The PCR program consisted of an initial denaturation at 95 °C for 20 s, followed by 40 cycles of 95 °C for 1 s and 60 °C for 20 s. The amount of RNA bound to NSP12 was quantified using amplification curves. Absolute RNA concentrations were calculated by interpolating Ct values from a standard curve generated using serial dilutions of the RNA library.

### 4.6. RNA-Protein Pull-Down Assay

A total of 60 fmol of RNA was denatured at 95 °C for 5 min, rapidly cooled on ice for 5 min, and incubated with 6 pmol of NSP12(P323L/G671S) protein, 2 µg of yeast tRNA (Sigma-Aldrich, St. Louis, MO, USA), 0.3 µL of RNase inhibitor, and 200 µL of SELEX buffer at room temperature for 20 min. The reaction mixture was then incubated with pre-washed Ni-NTA magnetic beads (Thermo Fisher Scientific, Waltham, MA, USA) for an additional 20 min at room temperature with gentle tapping. Beads were collected using a magnetic rack after brief centrifugation (13,000 rpm), and non-specifically bound RNAs were removed by washing twice with 400 µL of SELEX buffer. Bound RNA was eluted by adding 20 µL of DEPC-treated water and 16 µL of phenol, vortexing, and centrifuging at 13,000 rpm for 10 min. The aqueous phase (16 µL) was collected, mixed with an equal volume of 2× RNA loading dye, denatured at 95 °C for 5 min, and cooled on ice for 5 min. Samples were resolved on an 8% denaturing polyacrylamide gel containing 7 M urea at 180 V for 1 h. The gel was then transferred onto a 0.45 µm nylon membrane (MilliporeSigma, Burlington, MA, USA) at 200 mA for 1 h in 0.25× TBE buffer at 4 °C and UV cross-linked. For detection, the membrane was incubated in NorthernMax blocking buffer (Ambion, Thermo Fisher Scientific, Waltham, MA, USA) at 40 °C for 1 h, followed by overnight hybridization at 40 °C with 10 pmol of a biotin-labeled probe (5′-GGGGGGATCCATCGACCTCT GGGTTATG-3′) synthesized by Bioneer (Daejeon, Republic of Korea). The membrane was then washed for 6 × 5 min with Ambion washing buffer, incubated for 30 min with streptavidin-HRP (Novex, Thermo Fisher Scientific, Waltham, MA, USA) in blocking buffer, and rewashed six times. Detection was performed using ECL solution (Cytiva, Marlborough, MA, USA), and signals were visualized using an Amersham™ ImageQuant™ 800 imaging system (Cytiva, Marlborough, MA, USA).

### 4.7. Competition Assay

5′-Biotin-labeled RNA aptamers were synthesized by in vitro transcription using PCR-amplified DNA templates. For 2′-F RNA aptamers, 1 µg of PCR-amplified DNA was transcribed in a 20 µL reaction mixture containing 5 mM each of 2′-OH ATP, 2′-F UTP, and 2′-F CTP with 2 mM 2′-OH GTP and 10 mM 5′-biotin-GMP in 10× transcription buffer containing 10 mM DTT and 1 µL of T7 RNA polymerase (DuraScribe^®^ T7 Transcription Kit, LGC Biosearch Technologies, Novato, CA, USA). The reaction mixture proceeded at 37 °C for 16 h. For 2′-OH RNA aptamers, 1 µg of PCR-amplified DNA was transcribed in a reaction mixtue containing 75 mM of ATP, UTP, and CTP, and 37.5 mM GTP, 10 mM 5′-biotin-GMP, and T7 enzyme mix (MEGAscript™ T7 Transcription Kit, Thermo Fisher Scientific, Waltham, MA, USA). The mixture was incubated in a 37 °C for 3 h. In competition assays, 60 fmol of biotin-labeled RNA aptamer was incubated with 6 pmol of NSP12(P323L/G671S), 2 µg of yeast tRNA, 200 µL of SELEX buffer, and unlabeled competitor RNA (either the same aptamer RNA or a non-specific RNA library) at 2×, 20×, and 200× molar excess. Binding reactions were performed at room temperature for 30 min. Subsequent pull-down, washing, and detection procedures were conducted as described in Section 4.6.

### 4.8. Dissociation Constant (Kd) Analysis

To determine the dissociation constant (Kd), a series of NSP12(P323L/G671S) protein concentrations (0.6 nM, 1.8 nM, 5.4 nM, 16.2 nM, 48.6 nM, 145.8 nM, and 437.4 nM) were incubated with a fixed concentration of the aptamer. Binding reactions and subsequent procedures were performed as described in Section 4.5. The amount of RNA bound to NSP12(P323L/G671S) was quantified by real-time PCR, and the Kd value was calculated using GraphPad Prism 5.0.

### 4.9. Primer Extension Assay

Self-priming substrate RNA (5′-[FAM]UUUUCAUGCUACGCGUAGUUUUCUACGCG-3′) was synthesized by Bioneer (Daejeon, Republic of Korea) based on a sequence reported previously [39]. To prepare the substrate RNA, 100 pmol RNA was mixed with 10× annealing buffer (0.5 M KCl, 0.2 M HEPES) and adjusted to a final volume of 10 μL with DEPC-treated distilled water. The mixture was denatured at 75 °C for 5 min and then annealed by cooling slowly to 4 °C over an hour. For the formation of RdRp complex, 50 pmol of NSP12(P323L/G671S), 150 pmol of NSP7, and 150 pmol of NSP8 were incubated together for 1 h at 4 °C. Subsequently, 10 pmol of annealed substrate RNA was combined with the pre-formed RdRp complex, 5× extension buffer (0.5 M Tris-HCl, pH 8.0, 50 mM MgCl_2_, 50 mM DTT), and 50 pmol of RNA aptamer, and incubated for 5 min at 37 °C. Nucleotides were then added to a final concentration of 50 mM UTP, CTP, and GTP with 100 mM ATP and incubated for an hour at 37 °C. Total RNA was then extracted using phenol/chloroform, and RNA samples were mixed with 2× RNA loading dye, denatured at 95 °C for 5 min, cooled on ice, and loaded onto a 20% denaturing PAGE gel (1× TBE, 8 M urea). Electrophoresis was performed at 180 V for 2.5 h, and the gel was visualized using an Amersham™ ImageQuant™ 800 imager (Cytiva, Marlborough, MA, USA).

### 4.10. Mutagenesis

To generate the NSP12 double mutant (P323L/G671S), site-directed mutagenesis was performed using a commercial mutagenesis service provided by CosmoGenetech (Seoul, Republic of Korea). Single mutants of NSP12 (P323L or G671S) were obtained by site-directed mutagenesis using 20 ng of wild-type NSP12 construct as the template, along with 10 pmol of mutation-specific primers. P323L primers were 5′-AGCACCGTTTTTCCGCTTACCAGCTTTGGTCCG-3′ (forward) and 5′-CGGACCAAAGCTGGTAAGCGGAAAAACGGTGCT-3′ (reverse), and the G671S primers were 5′-ATGGTTATGTGTGGTAGTAGCCTGTATGTTAAA-3′ (forward) and 5′-TTTAACATACAGGCTACTACCACACATAACCAT-3′ (reverse). PCR amplification was carried out using 2× CloneAmp HiFi DNA polymerase (Takara Bio, Shiga, Japan) under the following conditions: 98 °C for 30 s, 60 °C for 30 s, and 72 °C for 1 min, for 35 cycles. The PCR products were treated with 20 units of DpnI restriction enzyme (Enzynomics, Daejeon, Republic of Korea) for an hour at 37 °C. The digested products were then transformed into *E. coli* DH5α competent cells, and plasmids were isolated using a mini-prep and verified by Sanger sequencing.

### 4.11. Statistics

Statistical analysis was performed using a two-tailed unpaired Student’s *t*-test (GraphPad Prism 5.0). Results are presented as means ± SDs from three independent experiments. Statistical significance was accepted at *p*-value < 0.05.

## Figures and Tables

**Table 1:** Selected aptamer sequence. Aptamer groups were classified based on sequence similarity and predicted secondary structure. The number of identical clones in each group is indicated in parentheses. Within each group, a representative sequence is shown, with identical regions denoted by hyphens (-) and variations from the representative highlighted in red.

Group	Selected Sequence of 2′-F RNA Pools
CV-F1 (3)	5′-CCTTACTATAATCTCTACGCTTATCTTATAGTACTGACCCAC-3′
CV-F2 (27)	5′-CCAATACTGTCATATTTTGGAATTGTTATGGAACCGCTATTC-3′
CV-F3 (2)	5′-CCTTTATGTGTTGTTACTCTCGATTGTTGAATATTTTTGCCTC-3′
CV-F4 (2)	5′-CCCTTTATCATAGTCCCAGATTAAGTGATAACTGTAGTCCAC-3′
CV-F5-1 (1) CV-F5-2 (1)	5′-CCGTTAAGTATTCACCTCAGCTATCATTAACCTGCTTTTAGT-3′ 5′--------------T-------------------------------C-------------------CTAC-------------3′
Group	Selected sequence of 2′-OH RNA pools
CV-OH1 (16)	5′-CCTTGAAGTCTTTGGGACTAGCTTCACGTACACGTCTCGA-3′
CV-OH2-1 (5) CV-OH2-2 (2)	5′-CCTTGAAGTCTTCGGGACTAGCTTCATGTACACGTATCGA-3′ 5′------------------------------------------------------T-------------------T----------3′
CV-OH3-1 (2) CV-OH3-2 (2) CV-OH3-3 (1) CV-OH3-4 (1) CV-OH3-5 (1)	5′-CCTTGAAGTCTTTGGGACTAGCTTCACGTACACGTATCGA-3′ 5′--------------------------C--------------------------CT-----T--------CC----------3′ 5′--------------------------C--------------------------CT-----------------------------3′ 5′--------------------------C-----------------------------------T--------CC----------3′ 5′--------------------------C--------------------------CT-----T----------C----------3′
CV-OH4 (1)	5′-CCTTGAAGTCTTCGGGACTAGCTTCTTGTACACGTCTCGA-3′

## Data Availability

The original contributions presented in this study are included in the article. Further inquiries can be directed to the corresponding author.
